# Spike suppression in a local cortical circuit induced by transcranial magnetic stimulation

**DOI:** 10.1007/s10827-012-0392-x

**Published:** 2012-05-16

**Authors:** Yoichi Miyawaki, Takashi Shinozaki, Masato Okada

**Affiliations:** 1 National Institute of Information and Communications Technology, Kyoto, Japan; 2 ATR Computational Neuroscience Laboratories, 2-2-2 Hikaridai, Seika-cho, Soraku-gun Kyoto, 619-0288 Japan; 3 Present Address: Center for Frontier Science and Engineering, The University of Electro-Communications, 1-5-1 Chofugaoka, Chofu, Tokyo 182-8585 Japan; 4 National Institute of Information and Communications Technology, Hyogo, Japan; 5 Graduate School of Frontier Sciences, The University of Tokyo, 5-1-5 Kashiwanoha, Kashiwa, Chiba 277-8561 Japan; 6 ERATO, Okanoya Emotional Information Project, Japan Science Technology Agency, Saitama, Japan; 7 RIKEN Brain Science Institute, Saitama, Japan

**Keywords:** Transcranial magnetic stimulation, Visual cortex, Suppression, Spiking neuron, Computational model

## Abstract

Transcranial magnetic stimulation (TMS) noninvasively interferes with human cortical function, and is widely used as an effective technique for probing causal links between neural activity and cognitive function. However, the physiological mechanisms underlying TMS-induced effects on neural activity remain unclear. We examined the mechanism by which TMS disrupts neural activity in a local circuit in early visual cortex using a computational model consisting of conductance-based spiking neurons with excitatory and inhibitory synaptic connections. We found that single-pulse TMS suppressed spiking activity in a local circuit model, disrupting the population response. Spike suppression was observed when TMS was applied to the local circuit within a limited time window after the local circuit received sensory afferent input, as observed in experiments investigating suppression of visual perception with TMS targeting early visual cortex. Quantitative analyses revealed that the magnitude of suppression was significantly larger for synaptically-connected neurons than for isolated individual neurons, suggesting that intracortical inhibitory synaptic coupling also plays an important role in TMS-induced suppression. A conventional local circuit model of early visual cortex explained only the early period of visual suppression observed in experiments. However, models either involving strong recurrent excitatory synaptic connections or sustained excitatory input were able to reproduce the late period of visual suppression. These results suggest that TMS targeting early visual cortex disrupts functionally distinct neural signals, possibly corresponding to feedforward and recurrent information processing, by imposing inhibitory effects through intracortical inhibitory synaptic connections.

## Introduction

Neural activity represents information about our perception and behavior. One effective method for investigating the relationship between neural activity and such functions is to manipulate neural activity and assess effects on the functions. Experimental approaches based on lesioning, pharmacological treatment, and electrical stimulation provide direct methods of manipulating neural activity in the brain, allowing the examination of consequent changes in perception and behavior (Salzman et al. 1990; Parker and Newsome 1998; Hupe et al. 1998). These techniques are typically invasive and difficult to apply to investigations of the healthy human brain. Transcranial magnetic stimulation (TMS) is a unique tool enabling human brain function to be disrupted noninvasively using a magnetic pulse produced by a coil placed on the scalp (Barker et al. 1985; Walsh and Cowey 2000). These advantages have led to TMS being widely used for examining the relationship between sites of stimulation and cortical functions of interest of the human brain. However, the fundamental neural mechanism of TMS-induced neural disruption remains largely unknown. This lack of understanding may lead to misinterpretation of observed experimental results, severely limiting the range of application of TMS.

**Fig. 1:**
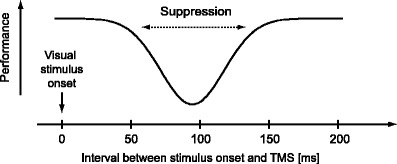
Visual stimulus suppression by TMS. In typical experiments, TMS is applied at various timings relative to the onset of visual stimulus presentation, and task performance (e.g., identification of a visual stimulus, typically presented briefly [tens of milliseconds]) is measured. Previous experiments have reported that task performance significantly deteriorates if TMS is applied during a limited time window, approximately from 50 ms to 150 ms (or even later timing) after the onset of stimulus presentation. The magnitude of suppression depends on the timing of TMS application, showing an inverted bell-shaped curve as illustrated. Suppression is typically most prominent around 80–100 ms

For example, TMS experiments targeting early visual cortex have shown puzzling observations regarding the suppression of visual perception. TMS suppresses perception of presented visual stimuli if applied around the occipital pole, to which early visual cortex is the closest area, with about 50 ms to 150 ms or even longer delay after stimulus presentation (Fig. 1) (Amassian et al. 1989; Kammer and Nusseck 1998; Kamitani and Shimojo 1999). A simple account for this observation is that TMS directly suppresses the activity of each individual neuron involved in feedforward propagation of visual stimulus information by electromagnetic interference. However, this account cannot explain TMS-induced suppression with a longer delay in the TMS experiments, because most neurons in early visual cortex begin spiking activity from approximately 60 ms after stimulus presentation, and reduce activity at around approximately 100 ms in response to feedforward sensory afferent input (Schmolesky et al. 1998). Thus, mechanisms other than direct suppression of feedforward neural activity may be also involved in TMS-induced visual suppression (Boyer et al. 2005; Lamme 2006; Koivisto et al. 2010).

Another candidate explanation for TMS-induced interference is based on interaction between stimulated neurons (Miyawaki and Okada 2004a, b). Since the spatial extent of the magnetic field induced by TMS is larger than the spatial scale of single neurons, a large number of neurons under the coil can be stimulated simultaneously. Neurons at nearby locations are likely to be mutually connected by excitatory and inhibitory synapses, and the TMS-induced effect on each individual neuron may influence other neurons through synaptic connections. Thus, interaction at the neural population level may explain experimental observations.

A possible approach for investigating the neural mechanisms of TMS is to observe how TMS affects neural activity. Recent electrophysiological studies using animals have revealed that visual cortical neurons change spike-firing patterns following TMS (Allen et al. 2007; Moliadze et al. 2003; Aydin-Abidin et al. 2006; de Labra et al. 2007). Human electroencephalography (EEG) studies have also shown that the time course of event-related potential changes after TMS (Thut et al. 2003; Jing et al. 2001) and oscillatory patterns can be entrained by rhythmic TMS (Thut et al. 2011). However, the mechanisms of this interference remain unclear, particularly regarding the involvement of interactions between stimulated neurons.

In this paper, we present an approach using a computational model to investigate the neural mechanisms of TMS-induced interference. In particular, we focused on TMS-induced suppression of visual perception, and analyzed a model of a local circuit exhibiting orientation selectivity in early visual cortex. A previous computational study (Miyawaki and Okada 2004a, b) analyzed TMS-induced interference on a local cortical circuit, but it was based on limited analyses of the equilibrium state of an idealized analog neuron network model that did not represent spiking membrane dynamics of realistic neurons. Here we used the model consisting of biologically realistic spiking neurons with Hodgkin–Huxley-type membrane dynamics, which were connected with excitatory and inhibitory synapses exhibiting feature selectivity similar to orientation tuning in early visual cortex. We then examined how spiking activity was perturbed by TMS applied to the local circuit model. Using this approach, we quantitatively analyzed TMS-induced interference with neural activity, particularly regarding the involvement of synaptic interaction and feedback input, while manipulating synaptic weights and afferent input properties.

## Methods

### Model of orientation-selective local cortical circuit

**Fig. 2:**
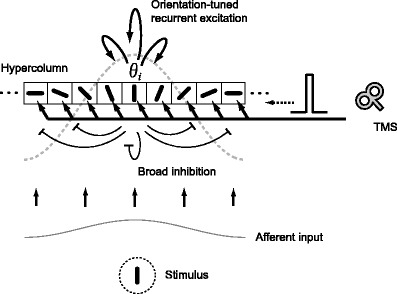
A neural network model of a local cortical circuit and TMS. The model represents a hypercolumn in early visual cortex, showing an orientation tuning function. The model consists of multiple neurons, each of which exhibits an orientation preference in a range of −90 to 90 degrees. The neurons are connected by excitatory and inhibitory synapses unless otherwise stated. Synaptic connections have a Mexican-hat-like structure (*gray dashed line*). The excitatory synapses exhibit cosine-type modulation depending upon differences in orientation preferences, and inhibitory synapses are uniformly distributed. Thus, neurons with similar orientation preferences excite each other, whereas all the neurons inhibit each other. Afferent input encodes stimulus orientation by firing rate. TMS is modeled as an excitatory current input with a short duration. Since the spatial extent of the TMS-induced magnetic field is significantly larger than the spatial scale of the cortical hypercolumn, TMS is assumed to stimulate all cortical neurons uniformly

We modeled a hypercolumn in early visual cortex, in which each neuron has a response preference to visual stimuli of a specific orientation (Fig. 2). Neurons were connected to each other by excitatory and inhibitory synapses. Each neuron was represented by a single-compartment conductance-based model. The membrane potential of *i*-th neuron at time *t*, *V*_*i*_(*t*), was described as,

$$ {C_m}\frac{{d{V_i}(t)}}{{dt}} = - I_i^m(t) + I_i^{{syn}}(t) + I_i^{{aff}}(t), $$

where $I_i^m(t)$ is the sum of Na, K, and leak current, and *C*_*m*_ is the membrane capacitance. $I_i^{{syn}}(t)$ represents the synaptic input current, which is the sum of excitatory and inhibitory currents from neurons in the hypercolumn. $I_i^{{aff}}(t)$ represents the current induced by the afferent input evoked by visual stimulation. See Appendix for detailed parameters of each ion current model.

Here we considered Mexican-hat-like synaptic connections, by which excitatory synapses provide strong connections between neurons with similar orientation preferences, whereas inhibitory synapses are distributed irrespective of orientation preferences. The excitatory and inhibitory synaptic conductance between *i*-th and *j*-th neuron, $G_{{ij}}^{\text{E}}$ and $G_{{ij}}^{\text{I}}$, were denoted as,

$$ \begin{array}{*{20}{c}}  {{\text{G}}_{{ij}}^{{\text{E}}} = {{J}_{{\text{E}}}}\left[ {1 + \cos 2\left( {{{\theta }_{i}} - {{\theta }_{j}}} \right)} \right],} \\  {G_{{ij}}^{{\text{I}}} = {{J}_{{\text{I}}}},} \\  \end{array}  $$

where *θ*_*i*_ and *θ*_*j*_ represent preferred orientation of *i*-th and *j*-th neuron. *J*_E_ and *J*_I_ are coefficients that determine the strength of excitatory and inhibitory synaptic connections, respectively. When the modeled synapses received a spike, synaptic conductance instantaneously changes by $G_{{ij}}^{\text{E}}$ and $G_{{ij}}^{\text{I}}$ for excitatory and inhibitory synapses, respectively, followed by exponential decay with a time constant of 5 ms. See Appendix for details of the synaptic input current model.

### Model of TMS-induced current

The effect of TMS on each neuron was modeled as an excitatory current pulse input. The electromagnetic effect of TMS on cortical neurons has been formulated using a realistic neuron model considering dendritic structure (Nagarajan et al. 1993; Kamitani et al. 2001), suggesting that the effect of TMS can be regarded as a transient current input to cortical neurons. Previous studies have also shown that pyramidal neurons are more susceptible to external magnetic fields than stellate cells (Kamitani et al. 2001). Thus, TMS may primarily induce an excitatory effect rather than an inhibitory one via excitatory cortical neurons. Based on this evidence, we assumed that each neuron received an excitatory pulse current input by TMS application.

In this paper, we assumed that all neurons in the model received an excitatory pulse current of the same amplitude (Fig. 2). Since the actual magnetic field induced by a TMS coil used in experiments has a specific profile with a spatial gradient, cortical neurons at different locations may receive TMS-induced effects of a different magnitude. However, the spatial scale of the neural population we consider here is as large as a single hypercolumn, and the variation of the TMS-induced magnetic field within the spatial scale can be negligible. Thus, the membrane potential equation for each neuron receiving a TMS-induced current can be described as,

$$ {C_m}\frac{{d{V_i}(t)}}{{dt}} = - I_i^m(t) + I_i^{{syn}}(t) + I_i^{{aff}}(t) + {I^{\text{TMS}}}(t), $$

where *I* ^*TMS*^(*t*) is the excitatory current input induced by TMS. *I* ^*TMS*^(*t*) was modeled as a pulse current of 30 *μ*A/cm^2^ with 1 ms duration.

### Model of afferent input

We used the following three types of afferent input models.

#### Broadly-tuned afferent input

The afferent input was represented by uncorrelated Poisson spike trains. The mean firing rate at time *t* to the *i*-th neuron, $f_i^{{aff}}(t)$, was described as,

$$ f_i^{{aff}}(t) = F^{{aff}}(t)\left[ {1 - \varepsilon + \varepsilon \cos 2\left( {{\theta_i} - {\theta_0}} \right)} \right] + {F^b}, $$

where *θ*_*i*_ is preferred orientation of the *i*-th neuron, *θ*_0_ is the presented stimulus orientation, *F* ^*aff*^(*t*) is the coefficient determining the maximum of the mean firing rate of the afferent input at time *t*, *ε* represents the degree of modulation of the afferent input strength depending upon difference between preferred orientation *θ*_*i*_ and presented orientation *θ*_0_, and *F* ^*b*^ is the amplitude of the background input. *ε* may have a value between 0 (all neurons receive afferent inputs with the same mean firing rate *F* ^*aff*^(*t*)) and 0.5 (a neuron with orientation preference *θ*_0_ receives *F* ^*aff*^(*t*) and that with *θ*_0_ + 90 degree receives 0). For a broadly-tuned afferent input model, we assumed that orientation tuning is not fully provided by the afferent input but is sharpened by intracortical recurrent interaction between neurons (Sompolinsky and Shapley 1997; Ringach et al. 1997; Shriki et al. 2003). We used *ε* = 0.175 as proposed by Shriki et al. (2003). We set *θ*_0_ = 0, but this does not compromise the generality of the model since the model is shift-invariant. The amplitude of the background input was set at *F* ^*b*^ = 100 Hz, slightly above the minimum value at which each individual neuron was able to initiate spikes, yielding very low background spike firing (<1 Hz).

The amplitude of *F* ^*aff*^(*t*) was changed as a parameter that reflects the contrast of presented visual stimuli. Note that *F* ^*aff*^(*t*) does not represent the spike input from a single afferent fiber but does represent the sum of a spike input volley from the multiple afferent fibers terminating at each neuron.

Experiments on TMS-induced visual suppression typically use a very short (less than 40 ms) duration of visual stimulus presentation. Accordingly, we set the duration of the afferent input to the model at 40 ms. Hence, the time course of *F* ^*aff*^(*t*) consisted of a transient 40-ms pulse unless otherwise stated (see Fig. 4, bottom row).

In the experiments, the origin of the time point was set at the onset of visual stimulus presentation as shown in Fig. 1, not the timing when the sensory afferent input arrived to early visual cortex. To compensate the absolute timing difference between the experiments and the model simulation, we have to take into account the conduction delay of spiking activity from the retina to early visual cortex. Here we shifted the origin of the time point so that the onset latency of spiking activity in the model matched the typical onset latency of neural activity of the primary visual cortex reported in animal and human electrophysiological experiments (66 ms) (Schmolesky et al. 1998; Yoshor et al. 2007). Note that given the background input of 100 Hz, neurons in the model took 13 ms on average to generate the spiking activity after the onset of the broadly-tuned afferent input, and thus the total amount of shift was 53 ms. This timing shift in the model simulation only changed the absolute timing of results.

#### Narrowly-tuned afferent input

To examine the possibility of TMS-induced interference without synaptic connections, we tested a model that comprises synaptically-unconnected neurons and afferent inputs that can provide orientation-tuned spiking activity even without synaptic connections.

We used the following functional form to represent the narrowly-tuned afferent input,

$$ \begin{array}{*{20}{c}}  {f_{i}^{{aff}}(t) = {{F}^{{aff}}}(t)h\left[ {\exp \left( { - \frac{1}{2}{{{\left( {\frac{{{{\theta }_{i}} - {{\theta }_{0}}}}{{{{\theta }_{s}}}}} \right)}}^{2}}} \right)} \right] + {{F}^{b}},} \\  {h(x) = \left\{ {\begin{array}{*{20}{c}}   x \hfill & {\left( {x \geqslant 0} \right)} \hfill  \\   0 \hfill & {\left( {x > 0} \right)} \hfill  \\  \end{array} ,} \right.} \\  \end{array}  $$

where *θ*_*s*_ is a parameter that specifies tuning width of the afferent input, *h*(*x*) is a threshold function, and *F* ^*b*^ is the amplitude of the background input. The amplitude of the background input was set at the same value as for the broadly-tuned afferent input model (*F* ^*b*^ = 100 Hz). With this tuned afferent input model, recurrent synaptic connections are not necessary to produce orientation-tuned spiking activity similar to that produced by the synaptically-connected model with broadly-tuned afferent input.

To determine parameters *F* ^*aff*^ and *θ*_*s*_, which produce similar orientation tuning functions to those produced by the synaptically-connected model with broadly-tuned afferent input, we first determined *F* ^*aff*^ that produced an orientation tuning function best fitted the synaptically-connected model for a specified amplitude of the afferent input in the range of 400 to 1,000 Hz with 100 Hz step (duration fixed at 40 ms) while keeping *θ*_*s*_ constant. We then determined *θ*_*s*_ that minimized the sum of square differences between orientation tuning functions of the connected model and the unconnected model for the searched amplitude range, by varying *θ*_*s*_ in the range of 12–21 degree with 1 degree step. Thus, the afferent input with the fitted *F* ^*aff*^ and *θ*_*s*_ produced orientation tuning functions in the unconnected model that were similar to those of the corresponding afferent input in the synaptically-connected model. The fitted value of *θ*_*s*_ was 16 degree.

#### Sustained excitatory input

Previous animal experiments have reported weak sustained neural activity in early visual cortex (Zipser et al. 1996; Lee et al. 1998). The findings of these studies suggest that the sustained neural activity may contain excitatory feedback signals from the higher visual cortex. To represent this component, we considered afferent input containing a weak sustained component followed by a transient component. In this case, the functional form of the afferent input was the same as the broadly-tuned afferent input, but *F* ^*aff*^(*t*) consisted of two components: $F_t^{{aff}}(t)$, a transient component representing feedforward afferent signals, and $F_s^{{aff}}(t)$, a weak sustained component representing excitatory input from other cortical areas. The background input was provided in the same way as for the above two afferent input models.

### Synaptic weights and stability of the model

The ratio of coefficients of excitatory synaptic strength *J*_E_ and inhibitory synaptic strength *J*_I_ plays a critical role in determining the spike firing patterns and stability of the model. These parameters should be determined so that the spike firing pattern and the stability of the model conform to properties of a typical local circuit in early visual cortex. Conventionally, local circuits in early visual cortex, particularly V1, are not thought to exhibit hysteresis between input and output firing rate. Thus the firing rate is expected to decrease to almost zero after the afferent input is terminated. On the other hand, recent studies suggest that neurons in early visual cortex show sustained activity produced by recurrent excitatory synaptic connections within early visual cortex (Lee et al. 1998; Bringuier et al. 1999). Such properties of neural firing can be represented by a local circuit model with recurrent excitatory connections showing weak hysteresis. Here we tested the above two cases with respect to synaptic weights, showing either no hysteresis or weak hysteresis, as local circuit models of early visual cortex.

**Fig. 3:**
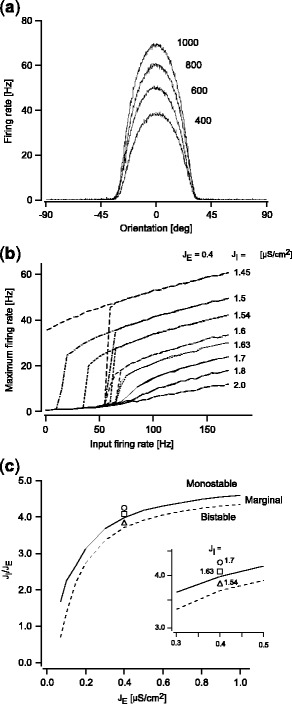
Spike firing patterns and synaptic weights. (**a**) Examples of spike firing patterns of the model with excitatory and inhibitory synaptic connections (*J*_E_ = 0.4, *J*_I_ = 1.7). The model was driven by the afferent input, whose maximum firing rate, *F* ^*aff*^(*t*), was kept constant over time. The firing rate for each neuron was measured after the firing pattern became stable, and five trials for afferent inputs of the same amplitude but with independent Poisson spike sequences were averaged. Representative examples from four different amplitudes are shown. (**b**) Relationship between input and output spike firing rate of the model. The maximum firing rate in the model is plotted against the amplitude of the afferent input (five trials were averaged as in panel (a)). To evaluate stability and hysteresis of the model, the amplitude of the afferent input was gradually increased from a subthreshold to a suprathreshold regime, then decreased in the opposite direction. Results are shown for representative examples of synaptic weight sets. (**c**) A phase diagram of the model with respect to synaptic weights. The phase diagram is parameterized by the excitatory synaptic weight *J*_E_ and the ratio of the inhibitory synaptic weight to the excitatory synaptic weight (*J*_I_/*J*_E_). The solid line and dashed line indicate the boundary between the monostable and marginal regime, and that between the marginal and the bistable regime, respectively. *Markers* indicate the representative synaptic weight sets used in the paper. The *inset* shows the area around the markers magnified

To determine appropriate synaptic weight parameters, we conducted pilot simulations using various synaptic weights and examined the stability of spike firing patterns. In these simulations, we used broadly-tuned afferent input with constant amplitude (i.e., *F* ^*aff*^(*t*) = const.) and quantified the spike firing pattern in the steady state (during 1,000–2,000 ms after the onset of the afferent input). Figure 3(a) shows the spike firing rate for afferent inputs with various amplitudes after the spike firing pattern became stable, with a particular synaptic weight set (*J*_E_ = 0.4, *J*_I_ = 1.7). The spike firing pattern shows clear tuning curves with peaks around the given stimulus orientation (*θ*_0_ = 0). The spike firing decreased to zero at approximately ± 30 degrees away from the given orientation, and the width was independent of the amplitude of the afferent input. These properties are consistent with typical orientation tuning curves shown in previous animal experiments and modeling studies (Ben-Yishai et al. 1995; Sompolinsky and Shapley 1997). Figure 3(b) plots the changes in peak firing rate of the tuning curve with the afferent input firing rate for different excitatory and inhibitory synaptic weights (*J*_E_ was fixed, and *J*_I_ was varied from 1.45 to 2.0). When *J*_I_ was large, the peak firing rate linearly increased with the afferent input firing rate if the afferent input firing rate was larger than the threshold (approximately 55 Hz) at which the network started to show spike firing rate higher than the background firing. When *J*_I_ was decreased, the peak firing rate suddenly increased around the threshold. When *J*_I_ became smaller than a certain value, spike firing remained for the subthreshold afferent input after the model became active, exhibiting hysteresis for the afferent input. When *J*_I_ was further decreased, the model kept spike firing even after the afferent input was terminated. Figure 3(c) delineates these three distinct parameter regimes in terms of synaptic weights: 1) a “monostable regime”, in which the model did not exhibit hysteresis between input and output spike firing rate and spike firing diminished immediately after the afferent input became a subthreshold level, 2) a “marginal regime”, in which the model exhibited weak hysteresis, and spike firing could be maintained for non-zero subthreshold afferent input, 3) a “bistable regime”, in which the model exhibited strong hysteresis and spike firing remained even after the afferent input was terminated. In this paper, we used synaptic weight parameters within the “monostable” and “marginal” regimes. Since neurons in early visual cortex are not thought to maintain stable firing patterns after visual stimuli have disappeared, we did not consider synaptic weights in the “bistable regime” for the following analyses.

### Numerical simulations

To examine effects of TMS on spiking activity, we performed numerical simulations while changing the timing of TMS application and the amplitude of the afferent input. Numerical simulations were performed with a total of 1,000 model neurons. The spiking activity of each neuron was calculated by solving differential equations using the fourth-order Runge–Kutta algorithm with a 0.05-ms time step.

The following models were tested to cover the possible variations of basic properties of local circuits in early visual cortex:

1. Model with synaptic connections (monostable regime)
  First, we tested a model with synaptic weights in the monostable regime (*J*_E_ = 0.4, *J*_I_ = 1.7), with broadly-tuned afferent input consisting of only a transient 40-ms component. The model only accounted for brief feedforward sensory afferent input.
2. Model without synaptic connections
  To examine the effect of synaptic connections in the model of TMS-induced interference with spiking activity, we tested a model without synaptic connections (*J*_E_ = *J*_I_ = 0). This model did not have a “network” structure. Rather, it constituted a collection of individual neurons, each of which had a preference for a specific orientation. Under these conditions, overall firing rate decreased and tuning curves became broad because of a lack of recurrent excitatory and inhibitory connections. For fair comparisons with the model with synaptic connections (Model 1), we adopted a narrowly-tuned afferent input model and adjusted the amplitude of the afferent input so that the output spike firing pattern matched that observed in the model with synaptic connections using the method described above (see section 2.3.2).
3. Model with synaptic connections (marginal regime)
  To examine effects of sustained activity in early visual cortex, we also tested three types of modification on Model 1. First, we decreased inhibitory synaptic weights relative to excitatory synaptic weights while using the same afferent input (broadly-tuned afferent input) as Model 1. This modification relatively increased recurrent excitation in the model, and spiking activity was prolonged even for the brief afferent input. Specifically, here we decreased *J*_I_ while *J*_E_ was fixed, and achieved a model with a higher ratio of excitatory to inhibitory synaptic weights compared to Model 1. Note that parameters were selected in the marginal regime (*J*_E_ = 0.4, *J*_I_ = 1.54), not the bistable regime.
4. Model with synaptic connections (monostable regime) and sustained excitatory input
  Second, we used a sustained excitatory input model as afferent input while the synaptic weights remained the same as in Model 1 (*J*_E_ = 0.4, *J*_I_ = 1.7). The amplitude of the sustained input was kept below the threshold (approximately 55 Hz) at which the network started to show spike firing rate higher than the background firing. This modification supplied additional excitatory current after the initial transient afferent input, as if excitatory feedback signals are supplied from other cortical areas. As a result, spiking activity was prolonged even if the amplitude of the sustained input was below the threshold of the spike firing. The sustained component was expressed by $F_s^{{aff}}(t)$, which was kept constant $\left( {F_s^{{aff}}(t) = 50\; Hz} \right)$ after the transient component $F_t^{{aff}}(t)$ was applied.
5. Model with synaptic connections of increased excitation (monostable regime) and sustained excitatory input
  Third, we combined modifications of synaptic weights and afferent input. In the marginal regime, there is an amplitude range for the sustained excitatory input in which the model can produce spike firing persistently (Fig. 3(b)). We avoided testing the model with such combinations of synaptic weights and sustained input, which would be inappropriate for a model of a local circuit in early visual cortex. Rather, we used a combination of synaptic weight (*J*_E_ = 0.4, *J*_I_ = 1.63) in the monostable regime but with recurrent excitation stronger than Model 1 and sustained input $\left( {F_s^{{aff}}(t) = 40\; \text Hz} \right)$ that produced spike firing that was prolonged relative to Model 1, but did not cause continuous spike firing.

### Evaluation of TMS-induced effect on spiking activity

The TMS-induced effect on spiking activity was evaluated as the total number of spikes remaining after TMS, which was then compared with a control condition without TMS. The synchronous spikes directly evoked by TMS were excluded from the count of spike numbers (specifically, spikes induced within 8 ms after the onset of TMS were excluded). The number of the residual spike count in the TMS condition was normalized by the spike count in the control condition. The onset timing of TMS was changed from −100 ms to 200 ms with a 1-ms step and from 200 ms to 400 ms with a 5-ms step relative to the afferent input onset, which corresponded to −47 to 453 ms after visual stimulation if conduction delay from the retina to early visual cortex is taken into account (see section 2.3.1 subsection). The normalized residual spike count was calculated for each TMS timing. We conducted five trials for each condition using different Poisson spike sequences with the same firing rate, and calculated the mean of the normalized residual spike counts and its standard error. We used this as a measure to quantify the magnitude of suppression, and compared it with task performance (e.g., the percentage of correct identification of presented visual stimuli) measured in previous TMS experiments targeting early visual cortex. As a representative example of experimental data, we used the results of Amassian et al. (1989). Although the residual spike count is not an identical measure to the measure of behavioral performance in the experiment, it is a suitable choice for comparison because it may reflect the amount of information in early visual cortex that influences the perception of presented visual stimuli.

We also used 1) the width of the effective time window for suppression and 2) the minimum value of the normalized spike count as indices to quantify the magnitude of suppression induced by TMS. The width of the effective time window for suppression was defined by the period during which the normalized residual spike count was less than 80 %. For comparisons with previous experimental data, we analyzed the results of Amassian et al. (1989) and quantified the width of the effective time window for suppression as the period during which percentage of correct responses was less than 80 %. Since the data were sparsely measured with 20 ms intervals in the experiment, we fitted inverted Gaussian functions and estimated the time at which performance crossed the 80 % value for each subject.

## Results

### Spike suppression by TMS for the model with synaptic connections

**Fig. 4:**
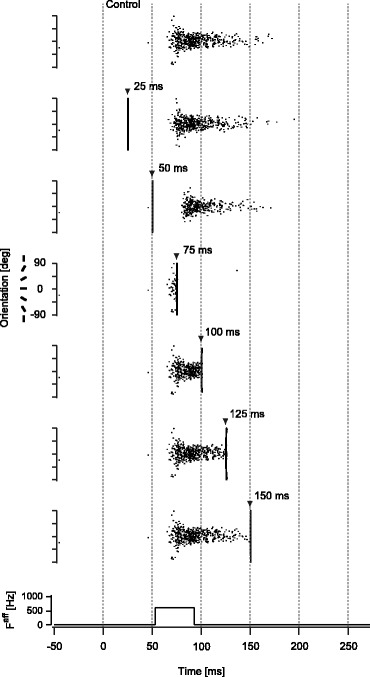
TMS-induced spike suppression for the model with synaptic connections. Examples of spike firing patterns in the synaptically-connected model (monostable regime (*J*_E_ = 0.4, *J*_I_ = 1.7)) with broadly-tuned afferent input (*F* ^*aff*^ = 600 Hz) are shown for representative timings of TMS application. Neurons in the model are aligned by the order of preferred orientation. Each dot represents the timing of the spike firing for a corresponding neuron. The *top row* illustrates the control condition without TMS. Triangle markers represent the onset of TMS. Since TMS induced synchronous spike firing in the model, the firing pattern appeared like a vertical line immediately after TMS application. The *bottom row* indicates the afferent input timing. Note that the time axis is shifted by 53 ms to compensate for conduction delay of the spiking activity from the retina to early visual cortex and to match the onset latency of spiking activity in the model with that measured in the experiment (see section 2.3.1)

We first tested Model 1, the synaptically-connected model (monostable regime; *J*_E_ = 0.4, *J*_I_ = 1.7) with broadly-tuned afferent input, which followed conventional notions of local circuit properties in early visual cortex. The model revealed spiking activity that was localized around neurons whose orientation preference was close to the presented stimulus orientation (*θ*_0_ = 0) (Fig. 4, top row). Spiking activity was transient since the afferent input lasted for only 40 ms and the synaptic weight was in the monostable regime.

TMS was found to suppress the spiking activity pattern if applied within a specific time window after the afferent input was given. Neurons in the model produced synchronized spiking activity immediately after TMS, irrespective of its timing. This spiking activity showed a primary effect of TMS, by which all neurons in the model were excited. Spike suppression followed this excitation. The magnitude of suppression induced by TMS varied with the timing of TMS application relative to the onset of the afferent input (Fig. 4, second to seventh rows). The spiking activity pattern vanished if TMS was applied at about 20 ms after the afferent input, which corresponds to 75 ms after visual stimulation if conduction delay from the retina to the cortex is taken into account (Fig. 4, fourth row). If the timing of TMS application was earlier or later, the effect of TMS was gradually reduced and spiking activity pattern was only partially suppressed.

**Fig. 5:**
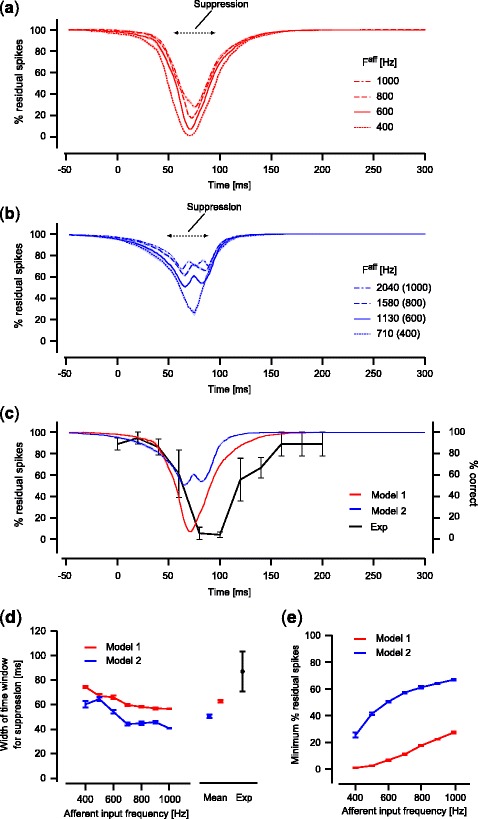
Relationship between magnitude of suppression and TMS application timing. (**a**) The magnitude of spike suppression induced by TMS for the synaptically-connected model (Model 1; monostable regime (*J*_E_ = 0.4, *J*_I_ = 1.7)). The horizontal axis corresponds to the TMS timing relative to the onset of the afferent input. Results show representative examples using the afferent input of four different amplitude values. Each line indicates a mean of five trials for each amplitude condition. Shaded areas around the lines indicate standard error. (**b**) The magnitude of spike suppression induced by TMS for the model without synaptic connections (Model 2; *J*_E_ = *J*_I_ = 0). Numbers in parentheses indicate the amplitude of the afferent input with which Model 1 produces a similar spike firing pattern. For example, the afferent input with *F* ^*aff*^ = 1130 Hz for Model 2 serves equivalently as the afferent input with *F* ^*aff*^ = 600 Hz for Model 1 in terms of observed spike firing patterns. (**c**) Comparisons of the magnitude of suppression between the models and the experiment. The experimental result of Amassian et al. (1989) is replotted as a black line, showing the percentage of correct responses for the letter identification task (*right axis*; mean of three subjects; *error bars*, standard error; data from Amassian et al. 1989). The percentage of residual spikes after TMS (*left axis*) is shown for Model 1 (*red line*) and Model 2 (*blue line*). Results of *F* ^*aff*^ = 600 Hz for Model 1 and *F* ^*aff*^ = 1130 Hz for Model 2 are shown as representative examples. (**d**) The width of the time window effective for suppression. Averaged values for five trials are plotted against the amplitude of the afferent input for Models 1 and 2 (*red and blue lines*, respectively; *error bars*, standard error). A total mean for tested amplitudes of the afferent inputs is also shown for each model to compare with the effective time window calculated from the experimental data of Amassian et al. (1989; *black markers*; mean of three subjects; *error bars*, standard error). (**e**) The minimum value of the normalized residual spikes count. Averaged values for five trials are plotted against the amplitude of the afferent input for Models 1 and 2 (*red and blue lines*, respectively; *error bars*, standard error). For panel (**d**) and (**e**), the amplitude of the afferent input for Model 2 was plotted as the value corresponding to that for Model 1

To evaluate the relationship between the magnitude of suppression and the timing of TMS, we measured the number of residual spikes relative to a control condition without TMS (see section 2.6 for details) while TMS timing was systematically varied (Fig. 5). Our analyses revealed the following findings: first, the effective timing of TMS application for suppression was restricted within a time window between approximately 40 ms and 110 ms, showing an inverted bell-shaped function peaking at about 70–75 ms (Fig. 5(a)). Second, the effective time window for suppression decreased (Figs. 5(a) and (d)) and the number of residual spikes increased (Figs. 5(a) and (e)) with the intensity of the afferent input, while the inverted bell-shaped function remained similar.

These results reproduced essential features of TMS-induced visual suppression observed in previous experiments. It was demonstrated that suppression was induced by TMS only if it was applied during a specific time window after visual stimulus presentation (Amassian et al. 1989; Kammer and Nusseck 1998; Kamitani and Shimojo 1999). It was also shown that the magnitude of suppression by TMS and the width of the time window effective for suppression decreased with the contrast of presented visual stimuli (Kammer and Nusseck 1998), which may increase the firing rate of afferent inputs to cortical neurons. These experimental observations qualitatively correspond to the current simulation results.

### Spike suppression by TMS for the model without synaptic connections

To examine the effect of synaptic connections on the TMS-induced spike suppression, we quantified the magnitude of TMS-induced spike suppression using Model 2, in which no synaptic connections exist and orientation-tuned responses are produced by narrowly-tuned afferent inputs.

**Fig. 6:**
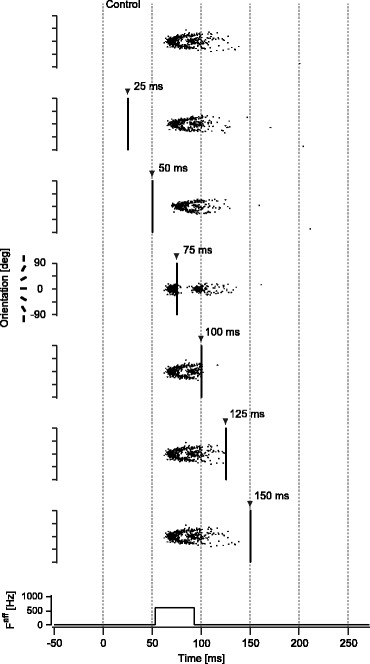
TMS-induced spike suppression for the model without synaptic connections. Examples of spike firing patterns in the model without synaptic connections (*J*_E_ = *J*_I_ = 0) receiving narrowly-tuned afferent input are shown for representative timings of TMS application in the same way as in Fig. 4. Presented results were obtained with an amplitude of *F* ^*aff*^ = 1130 Hz, by which the model without synaptic connections yielded spike firing patterns closest to those in Model 1 with *F* ^*aff*^ = 600 Hz in the control condition without TMS

For the model without synaptic connections, TMS suppressed spiking activity in a way that was similar to the original model with synaptic connections, except for the magnitude of spike suppression. Figure 6 shows spike raster plots of the model without synaptic connections, observed with various TMS application timings, as illustrated in Fig. 4. TMS was also able to suppress the spiking activity patterns if applied within a specific time window relative to the onset of the tuned afferent input. The timing at which TMS was most effective for suppression was also approximately 20 ms after the onset of the afferent input, corresponding to 75 ms after visual stimulation if conduction delay from the retina to the cortex is taken into account (Fig. 6, fourth row). The magnitude of suppression for this model, however, was significantly reduced compared to that for the model with synaptic connections. For example, even at the most effective timing, spikes remained after TMS application (Fig. 6, fourth row). Quantitative analyses showed that the magnitude of TMS-induced suppression significantly decreased (i.e., residual spikes increased; Figs. 5(b) and (e); two-way ANOVA, *p* < 0.05), and the time window effective for suppression were also significantly reduced compared to the original model with synaptic connections (Figs. 5(b) and (d); two-way ANOVA, *p* < 0.05). These results indicate that TMS exerted a much stronger inhibitory effect in the model with synaptic connections compared to the model without synaptic connections.

### Quantitative comparison with experimental results

Our results revealed that TMS suppressed spiking activity in a local cortical circuit model. In addition, suppression was induced only when TMS was applied within a restricted time window relative to the afferent input onset. Although these results were qualitatively consistent with the typical experimental data, there was an inconsistency in the width of the time window effective for suppression. Figure 5(c) replots the representative experimental data from a previous study (Amassian et al. 1989), showing the magnitude of visual suppression in terms of the correct rate of visual target identification. The suppressive effect of TMS started around 40 ms, exhibiting the largest effect at about 80–100 ms, and continued up to 200 ms after visual stimulus onset. Other experiments have reported similar results in terms of the effective time window for suppression (Kammer and Nusseck 1998; Kamitani and Shimojo 1999). Our simulation results revealed that suppression of the spiking activity began at about 40 ms and showed the largest effect at about 70–75 ms if conduction delay from the retina to the cortex is taken into account. The model prediction is thus in accord with the experimental results in the early time period after the onset of visual stimulation. However, suppression predicted by the model ended earlier than that reported in the experimental data (Fig. 5(c)). Quantitative analyses revealed that the width of the time window for suppression was significantly shorter in our simulation than in the experimental data (Fig. 5(d); *p* < 0.05 for difference between experimental data and the mean of simulation data, ANOVA). For the model without synaptic connections, the width of the time window effective for suppression was even shorter than in the model with synaptic connections (Fig. 5(d)). Thus, the models we tested can only partially explain experimentally observed TMS-induced suppression.

### Spike suppression in the models representing sustained neural activity

**Fig. 7:**
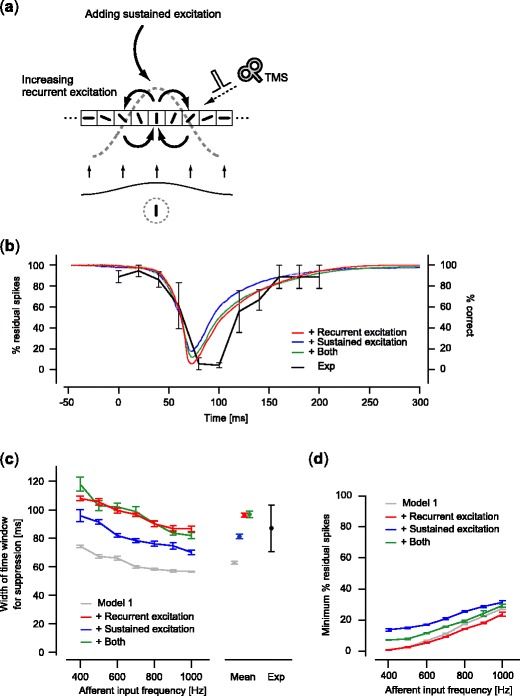
Relationship between magnitude of suppression and TMS timing for the models representing sustained neural activity. (**a**) Modified models representing sustained neural activity. Model 1 was modified by increasing the synaptic weight for recurrent excitation (Model 3–1), adding weak sustained excitatory input (Model 3–2), or applying both (Model 3–3). (**b**) The magnitude of suppression for the three models representing sustained neural activity. Results of *F* ^*aff*^ = 600 Hz were shown as representative examples for each model (mean of five trials; shaded area, standard error). Each of the colors corresponds to one of the three models. The experimental result (Amassian et al. 1989) is plotted as shown in Fig. 5(**c**). (**c**) The width of the time window effective for suppression. (**d**) The minimum value of the residual spikes. For panels (**c**) and (**d**), results are shown in the same way as in Fig. 5(**d**) and (**e**), respectively, for the three models and the experiment. Results of Model 1 are shown for comparison in panels (**c**) and (**d**)

To explain TMS-induced suppression in the late period, here we used modified models, or Model 3 s, which represented sustained neural activity induced by either recurrent excitation (Model 3–1), sustained excitatory input (Mode 3–2), or both (Model 3–3) (Fig. 7(a)). We tested whether these modified models revealed spike suppression that was quantitatively consistent with experimental data, particularly in the late period. Here we only showed the results using particular sets of parameters (Model 3–1: *J*_E_ = 0.4, *J*_I_ = 1.54, $F_s^{{aff}}(t) = 0\;Hz$; Model 3–2: *J*_E_ = 0.4, *J*_I_ = 1.7, $F_s^{{aff}}(t) = 50\; Hz$; Model 3–3: *J*_E_ = 0.4, *J*_I_ = 1.63, $F_s^{{aff}}(t) = 40\;Hz$; see Fig. 3(b) and (c) for firing properties and stability of the models using these parameter sets). Minor variations of these parameter choices did not change the following results qualitatively.

Our analyses revealed that all of these models showed suppression of the spiking activity in the late period, quantitatively consistent with the experimental data. Figure 7(b) shows the time course of the magnitude of suppression induced by TMS, calculated in the same way as in Fig. 5. The time course of the magnitude of suppression on visual perception measured in a previous experiment (Amassian et al. 1989) is also replotted, as in Fig. 5(c). All of the models better fit the experimental results in the late period than the original Model 1. The width of the effective time window for suppression significantly increased for the modified models relative to the original Model 1 (Fig. 7(c); Bonferroni-corrected *p* < 0.05 for multiple comparisons, two-way ANOVA), exhibiting values in a consistent range with the experimental results (Fig. 7(c); Bonferroni-corrected *p* > 0.05 for multiple comparisons for the difference between the experiment and the mean of each modified model, ANOVA). Although the spiking activity was suppressed similarly for all the modified models as in the original model, there were statistical differences in the number of the residual spike count after TMS depending on the model types (Fig. 7(d); Bonferroni-corrected *p* < 0.05 for multiple comparisons, two-way ANOVA). These results indicate that a model based on conventional assumptions about local circuits in early visual cortex is not sufficient; strong recurrent excitation and sustained excitatory input from other cortical areas, allowing the model to exhibit nonlinear hysteresis in spiking activity, should be also taken into account to reproduce the time course of experimentally observed TMS-induced suppression.

## Discussion

### Neural sources of TMS-induced suppression

In this paper, we examined TMS-induced suppression of spiking activity using a local circuit model of early visual cortex. Our results revealed that a TMS-like perturbation suppressed spiking activity if applied within a restricted time window after the onset of afferent input.

Although an intuitive assumption about the mechanisms behind the suppression of spiking activity is that each individual neuron is directly suppressed by the TMS-induced current, our results suggest that synaptic connections between neurons in the local cortical circuit also play a substantial role. Previous studies have shown that a magnetic pulse can suppress the spiking activity of individual cortical neurons using biophysically-realistic models (Kamitani et al. 2001; Miyawaki and Okada 2004a). However, our results revealed that a model without synaptic connections showed inhibitory effects that were significantly smaller than those in a model with synaptic connections. In the former model, the only source of suppression was the hyperpolarization current of each individual neuron, whereas the latter model also included inhibitory synaptic connections between neurons. The primary effect of TMS in our model is excitatory, but induced spiking activity can yield inhibitory effects through recurrent inhibitory synaptic connections. Indeed, a spike firing in a neuron in our model produced inhibitory postsynaptic potential to other neurons with a decay time constant of about 30 ms, which is within a range consistent with physiological data for GABAergic synapses in the visual cortex (Xiang et al. 1998, 2002). These results suggest that inhibitory synaptic interactions in the local cortical circuit also play an important role as a neural source of TMS-induced suppression.

Furthermore, the suppressive effect induced by successive TMS pulses could be difficult to explain by mechanisms at the single cellular level alone. A previous study reported that successive TMS pulses with an interval of more than 100 ms suppressed responses to visual stimuli even though each single pulse was not strong enough to induce suppression (Amassian et al. 1993). To induce such a superpositional effect, it may be necessary for the effect of each single weak TMS pulse to remain until the next TMS pulse. However, it seems unlikely given the electric and chemical properties of individual neurons, because the effect of each single weak TMS pulse would be refreshed within the membrane time constant.

Studies investigating the motor cortex using TMS have also suggested the involvement of inhibitory synaptic connections in TMS-induced effects. Prolonged inhibitory responses are observed on electromyography (EMG) after TMS applied to the motor cortex, which is known as the cortical silent period (Inghilleri et al. 1993; Pell et al. 2011). Priori et al. reported that hyperventilation, which reduces extracellular Ca^2+^ concentration and decreases GABAergic synaptic transmission, shortened the duration of the cortical silent period (Priori et al. 1995). Other studies have also reported that administration of GABAergic drugs modulated the amplitude of motor evoked potentials induced by TMS targeting the motor cortex (Inghilleri et al. 1993; Ziemann et al. 1996a, b). These experiments suggest that GABAergic inhibitory synaptic transmission may play an important role in TMS-induced interference with neural activity in the motor cortex. Since GABAergic inhibitory synaptic connections are ubiquitous in the cortex, including early visual cortex, it is likely that similar mechanisms based on GABAergic inhibitory synapses may be also involved in visual suppression induced by TMS targeting early visual cortex.

In this study, we assumed that each individual neuron was stimulated by an excitatory current induced by TMS, but axonal fibers could also be candidate sites of stimulation. Previous studies suggest that visual suppression induced by TMS is largely caused by stimulation of the bend of axonal fibers projecting to early visual cortex (Amassian et al. 1994; Kammer et al. 2005). However, our current simulation results are unlikely to change even if the TMS-induced effect is modeled as an excitatory current in axonal fibers rather than cortical neurons. Essential for TMS-induced suppression in our model is the simultaneous stimulation of multiple neurons with inhibitory synaptic connections. Thus, the model stimulating the axonal fibers projecting to early visual cortex and the model directly stimulating neurons in early visual cortex may work equivalently provided that a population of neurons in early visual cortex is activated.

Although we focused on changes in the spike firing rates of neurons in this study, TMS could also induce changes in spike synchronization in neuronal populations since it can stimulate many neurons simultaneously. A previous TMS experiment has shown that EEG activity was entrained by rhythmic TMS, suggesting that TMS can influence oscillatory neural activity (Thut et al. 2011). To investigate effects of TMS on spike synchronization and oscillatory activity systematically, it may be better to analyze steady states of a spiking neuronal population rather than transient states that were investigated in this paper. Systematic analyses of the relationship between TMS and spike synchronization and oscillatory activity remain a topic for future studies.

### Suppression of spiking activity and visual perception

Quantitative analyses of the width of the effective time window for suppression showed that a model without hysteresis in spike firing rate was insufficient. Either strong recurrent excitatory synaptic connections, weak sustained excitatory inputs, or both, were necessary for the simulation results to quantitatively match the experimental data, particularly for the late period after visual stimulation. These results suggest that non-linear properties with hysteresis in spike firing of the local cortical circuit may be critical for characterizing the temporal aspects of TMS-induced visual suppression.

These findings also imply that sustained excitation in early visual cortex might play an important role in yielding conscious visual perception, which might be mediated by recurrent excitatory synaptic connections in early visual cortex or excitatory feedback signals from higher visual areas (Zipser et al. 1996; Lee et al. 1998; Lamme and Roelfsema 2000; Bullier 2001). This suggests that visual suppression induced by TMS targeting early visual cortex might actually involve the suppression of two distinct neural signals: feedforward neural signals transferred in the early period, and delayed neural signals mediated by recurrent excitation or feedback input from the higher cortical areas in the late period.

Recent TMS studies have raised possibility of two distinct components in TMS-induced visual suppression (Boyer et al. 2005; Lamme 2006), consistent with our model predictions. TMS experiments typically use a subjective report measure to determine whether presented visual stimuli were consciously perceived, as a criterion of visual suppression. In contrast, Boyer et al. (2005) utilized a forced-choice paradigm to probe subconscious perception, demonstrating that TMS applied within the late period (>100 ms) suppressed conscious perception of the presented visual stimulus, but subjects were able to report correct answers when they were forced to guess the presented stimulus, as in blindsight patients. The same paradigm could be applied to probe visual suppression in the early period (<100 ms) to test whether these blindsight-like phenomena are observed as Boyer et al. (2005) showed in the late period (Lamme 2006). If the TMS-induced suppression in the early period is mediated by suppression of feedforward neural signal propagation, it is unlikely that subjects would be able to respond correctly, unless the feedforward signal bypasses V1 through the geniculo-extrastriate pathway. Koivisto et al. (2010) recently tested this hypothesis, showing that forced-choice performance of a visual discrimination task progressively increased with the timing of TMS application relative to stimulus presentation (Koivisto et al. 2010). These results support our model predictions and the hypothesis that TMS-induced visual suppression consists of two distinct components with respect to TMS timing relative to the onset of afferent sensory input.

### Application to other cortical areas

Although we focused on the local circuit model in early visual cortex, it may be possible to use our modeling framework to analyze TMS-induced effects on other cortical areas. Experimental evidence suggests that an intracortical inhibitory network, presumably mediated by GABAergic synapses, is involved in TMS-induced effects on motor cortical activity (Inghilleri et al. 1993; Priori et al. 1995; Ziemann et al. 1996a, b). Our framework using a network-based model could be useful for providing theoretical interpretations of experimental TMS data in the motor cortex. The dorsolateral prefrontal cortex (DLPFC), which plays an important role in spatial working memory (Funahashi et al. 1989), may be another interesting target for investigation with our model. Previous modeling studies suggest that the DLPFC encodes spatial working memory in neural populations with Mexican-hat-like synaptic connections (Compte et al. 2000). Such a network structure is identical to our model, except in terms of the magnitude of synaptic weights. Thus, our framework could be easily extended to predict TMS-induced effects in the DLPFC. This may improve understandings of neural representation of spatial working memory in human subjects.

It is also important to investigate whether the specific spatial patterns of synaptic connections are necessary to produce similar properties of spike suppression. In preliminary analyses, we observed that spatially-unstructured random synaptic connections showed spike suppression after TMS. Further analyses are necessary to systematically compare the effects of different spatial patterns of synaptic connections on the properties of spike suppression.

Our model could be useful for predicting differences in synaptic connection weights in target cortical areas by comparing temporal characteristics of TMS-induced suppression. As shown in the results, synaptic weights are critical in characterizing TMS-induced effects on spiking activity (Fig. 7). For example, if a target cortical area exhibits recurrent excitatory synaptic connections that are stronger than those in early visual cortex, we might expect that the width of the time window effective for TMS-induced suppression would increase. Thus, by combining model predictions and experimental observations, we might be able to estimate synaptic connectivity and the network structure of local cortical circuits of the human brain using TMS.

## Appendix

### Model of Hodgkin–Huxley-type neurons

Here we describe the mathematical details for the model of Hodgkin–Huxley-type neurons that constitute a cortical local circuit (see also Shriki et al. 2003).

The differential equation of the membrane potential of the *i*-th neuron at time *t*, *V*_*i*_(*t*), was described as (see also section 2),

$$ {C_m}\frac{{d{V_i}(t)}}{{dt}} = - I_i^m(t) + I_i^{{sy{n_{{all}}}}}(t), $$

where $I_i^m(t)$ is the sum of membrane ion current, and $I_i^{{sy{n_{{all}}}}}(t)$ represents the synaptic input current. *C*_*m*_ is the membrane capacitance and set as ${{C}_{m}} = 1\mu F/{\text{c}}{{{\text{m}}}^{2}}$.

#### Membrane ion current

$I_i^m(t)$ is the sum of the sodium (Na), potassium (K), and leak (L) currents,

$$ I_i^m(t) = I_i^{\text{Na}}(t) + I_i^{\text{K}}(t) + I_i^{\text{L}}(t), $$

where $I_i^{\text{Na}}(t) = {\bar{g}^{\text{Na}}}m_{\infty }^3h\left( {{V_i}(t) - {E^{\text{Na}}}} \right)$, $I_i^{\text{K}}(t) = {\bar{g}^{\text{K}}}n^4\left( {{V_i}(t) - {E^{\text{K}}}} \right)$, $I_i^{\text{L}}(t) = {g^{\text{L}}}\left( {{V_i}(t) - {E^{\text{L}}}} \right)$. The gating variables *x* (= *m*,*h*,*n*) are described by first-order kinetics, ${{{dx}} \left/ {{dt}} \right.} = {{{\phi \left( {{x_{\infty }} - x} \right)}} \left/ {{{\tau_x}}} \right.}$, where *ϕ* is the temperature coefficient and set as *ϕ* = 10. *x*_∞_ and *τ*_*x*_ are ${x_{\infty }} = {{{{\alpha_x}}} \left/ {{\left( {{\alpha_x} + {\beta_x}} \right)}} \right.}$ and ${\tau_x} = {{1} \left/ {{\left( {{\alpha_x} + {\beta_x}} \right)}} \right.}$, respectively, where ${\alpha_m} = {{{ - 0.1\left( {V + 30} \right)}} \left/ {{\left( {\exp \left( { - 0.1\left( {V + 30} \right)} \right) - 1} \right)}} \right.}$, ${\beta_m} = 4\exp \left( {{{{ - \left( {V + 55} \right)}} \left/ {{18}} \right.}} \right)$, ${\alpha_h} = 0.07\exp \left( {{{{ - \left( {V + 44} \right)}} \left/ {{20}} \right.}} \right)$, ${\beta_h} = {{1} \left/ {{\left( {\exp \left( { - 0.1\left( {V + 14} \right)} \right) + 1} \right)}} \right.}$, ${{\alpha }_{n}} =  - 0.01\left( {V + 34} \right)/\left( {\exp \left( { - 0.1\left( {V + 34} \right)} \right) - 1} \right)$, and ${\beta_n} = 0.125\exp \left( {{{{ - \left( {V + 44} \right)}} \left/ {{80}} \right.}} \right)$. The other constant parameters are as follows: maximum ion conductance, ${\bar{g}^{\text{Na}}} = 100\;{{\text{ mS}} \left/ {{{\text{c}}{{\text{m}}^{{2}}}}} \right.}$, ${\bar{g}^{\text{K}}} = 40\;{{\text{ mS}} \left/ {{{\text{c}}{{\text{m}}^{{2}}}}} \right.}$, and ${g^{\text{L}}} = 0.05{{\text{ mS}} \left/ {{{\text{c}}{{\text{m}}^{{2}}}}} \right.}$; and reversal potential, *E* ^Na^ = 55 mV, *E* ^K^ = −80 mV, and *E* ^L^ = −65 mV. The parameters were set according to Shriki et al. (2003).

#### Synaptic current

The synaptic current, $I_i^{{sy{n_{{all}}}}}(t)$, is further decomposed into recurrent synaptic input, $I_i^{{syn}}(t)$, and afferent synaptic input, $I_i^{{aff}}(t)$. The recurrent synaptic input, $I_i^{{syn}}(t)$, was expressed as excitatory and inhibitory currents from neurons in the local circuit. The afferent synaptic input, $I_i^{{aff}}(t)$, was expressed as excitatory current evoked by visual stimulation. Thus, the total synaptic current was described as,

$$ \begin{array}{*{20}{c}}  {I_{i}^{{sy{{n}_{{all}}}}}(t) = I_{i}^{{syn}}(t) + I_{i}^{{aff}}(t) = \sum\limits_{{j = 1}}^{N} {g_{{ij}}^{{\text{E}}}(t)\left( {{{E}^{{\text{E}}}} - {{V}_{i}}(t)} \right)} } \\  { + \sum\limits_{{j = 1}}^{N} {g_{{ij}}^{{\text{I}}}(t)\left( {{{E}^{{\text{I}}}} - {{V}_{i}}(t)} \right)}  + {{g}^{{aff}}}(t)\left( {{{E}^{{aff}}} - {{V}_{i}}(t)} \right),} \\  \end{array}  $$

where $g_{{ij}}^{\text{E}}(t)$ and $g_{{ij}}^{\text{I}}(t)$ are the excitatory and inhibitory synaptic conductance from *j*-th to *i*-th neuron; *g* ^*aff*^(*t*) is the excitatory synaptic conductance of the afferent input to *i*-th neuron; *E* ^E^, *E* ^I^, and *E* ^*aff*^ are the reversal potentials for their respective synapses; and *N*(= 1,000) is the number of neurons in the local circuit model. The differential equation of synaptic conductance was described as,

$$ \frac{{dg(t)}}{{dt}} = - \frac{{g(t)}}{{{\tau_{{syn}}}}} + G\sum\limits_{{{t_k}}} {\delta \left( {t - {t_k}} \right)}, $$

where *g*(*t*) is $g_{{ij}}^{\text{E}}(t)$, $g_{{ij}}^{\text{I}}(t)$, or *g* ^*aff*^(*t*), and *G* is $G_{{ij}}^{\text{E}}$, $G_{{ij}}^{\text{I}}$, or *G* ^*aff*^, respectively (see also section 2). $\delta \left( {t - {t_k}} \right)$ is one if *t* = *t*_*k*_, otherwise zero. $G_{{ij}}^{\text{E}}$ and $G_{{ij}}^{\text{I}}$ determine how neurons in the local circuit interact through synapses. We used a Mexican-hat-like interaction to represent orientation-tuning function in early visual cortex (Ben-Yishai et al. 1995; Shriki et al. 2003). The specific profile is described in section 2 in the main text. The other constants are set as follows: *E* ^E^ = *E* ^*aff*^ = 0 mV, *E* ^I^ = −80 mV, and *τ*_*syn*_ = 5 ms.
